# Perception of aging and changes in later life: Findings from ethnographic interviews with older adults

**DOI:** 10.1093/geroni/igaf122.685

**Published:** 2025-12-31

**Authors:** Lun Li, Yeonjung Lee

**Affiliations:** MacEwan University, Edmonton, Alberta, Canada; University of Calgary, Calgary, Alberta, Canada

## Abstract

Older adults’ understanding of aging and their perception of aging-related issues (e.g., health, social life) have fundamental impacts on their preparation for later life, and the quality of life during the aging process. This study intends to explore how older adults understand aging and aging-related issues through later life experiences during and after the COVID-19 pandemic. This study is based on 21 ethnographic interviews conducted by social work students who enrolled in a gerontology course in a western Canada university between 2022 and 2024. The participants of interviews are older adults aged 70 years and older. A thematic analysis was conducted to all 21 transcripts. Four main themes were identified, including 1) Older adults normalize the aging process through the changes of family/generation structure, a shift from a productive to a recreative social life, and the narrowed networking size; 2) Older adults adjust to the deterioration of physical health and functional capability using various strategies; 3) Older adults distinguish the perception of aging from the concept of oldness; and 4) The COVID-19 pandemic and recent developments (e.g., technology advancement) contribute to older adults’ challenges in navigating their later life. Findings suggest that older adults actively make changes to their different life aspects to enhance the quality of later life. It also reveals older adults’ understanding of aging process, particularly their acceptance of age but not oldness. This study further emphasizes the salience of aging-friendly society without barriers to enable healthy and active aging.

